# Structural and functional insights into *Escherichia coli* O32:H37 contact dependent inhibition

**DOI:** 10.1063/4.0001106

**Published:** 2025-10-27

**Authors:** Karolina Michalska, Lucy Stols, Dinh Quan Nhan, Fernando Garza-Sánchez, William H. Eschenfeldt, Christopher S. Hayes, Andrzej Joachimiak

**Affiliations:** 1 X-ray Science Division, Argonne National Laboratory, Lemont, IL, USA; 2 Center for Structural Biology of Infectious Diseases, Consortium for Advanced Science and Engineering, University of Chicago, Chicago, IL, USA; 3 Department of Molecular, Cellular and Developmental Biology, University of California, Santa Barbara, CA, USA; 4 Biomolecular Science and Engineering Program, University of California, Santa Barbara, CA, USA; 5 Department of Biochemistry and Molecular Biology, University of Chicago, Chicago, IL, USA

## Abstract

Many gram-negative bacteria utilize contact-dependent growth inhibition (CDI) systems to introduce toxic effector proteins into neighboring cells, thereby outcompeting them. In CDI^+^ strains, CdiA effector proteins, carrying C-terminal toxin (CT) domains, are delivered into target cells through receptor-mediated pathways. To prevent self-intoxication, these bacteria produce CdiI immunity proteins that bind and neutralize the CT domains. Here, we present the crystal structure of the CT•CdiIO32:H37 complex from Escherichia coli O32:H37. Our findings reveal that CTO32:H37 is structurally homologous to the C-terminal tRNase domain of colicin D, and contains similar catalytic centers. However, CTO32:H37 exhibits non- specific RNase activity, unlike colicin D, which targets the anticodon loops of tRNAArg isoacceptors. The CdiIO32:H37 immunity protein features a unique fold coordinating a central Fe3+ ion with cysteine residues. Intriguingly, CT• CdiIO32:H37 complex co-purifies with endogenous elongation factor Tu (EF-Tu), forming a stable ternary complex. Although CTO32:H37 does not stably interact with EF-Tu without CdiIO32:H37, EF-Tu is essential for RNase activity in vitro. AlphaFold3 modeling suggests that CTO32:H37 binds to the GTPase domain of EF-Tu at a site overlapping the EF-Ts binding site. Our data indicate that EF-Tu supports the toxin’s active site, stabilizing a core tryptophan residue that interacts with a catalytic histidine.

